# A 3-D evaluation of transverse dentoalveolar changes and maxillary first molar root length after rapid or slow maxillary expansion in children

**DOI:** 10.1590/2177-6709.24.3.079-087.oar

**Published:** 2019

**Authors:** Helder Baldi Jacob, Gerson Luiz Ulema Ribeiro, Jeryl D. English, Juliana da Silva Pereira, Mauricio Brunetto

**Affiliations:** 1 The University of Texas Health Science Center at Houston School of Dentistry, Department of Orthodontics (Houston/ TX, USA).; 2 Universidade Federal de Santa Catarina, Departamento de Ortodontia (Florianópolis/SC, Brazil).; 3 Private practice (Florianópolis/SC, Brazil).; 4 Private practice (Curitiba/PR, Brazil).

**Keywords:** Maxillary expansion, Root resorption, Cone beam computed tomography

## Abstract

**Objective:**

The objective of the present study was to conduct a randomized clinical trial comparing the effects of rapid maxillary expansion (RME) and slow maxillary expansion (SME). Maxillary permanent first molar root length and tooth movement through the alveolus were studied using cone-beam computed tomography (CBCT).

**Methods:**

Subjects with maxillary transverse deficiencies between 7 and 10 years of age were included. Using Haas-type expanders, children were randomly assigned to two groups: RME (19 subjects, mean age of 8.60 years) and SME (13 subjects, mean age of 8.70 years).

**Results:**

Buccal cortical, buccal bone thicknesses and dentoalveolar width decreased in both groups. In the RME group the greatest decrease was related to distal bone thickness (1.26 mm), followed by mesial bone thickness (1.09 mm), alveolar width (0.57 mm), and the buccal cortical (0.19 mm). In the SME group the mesial bone thickness decreased the most (0.87 mm) and the buccal cortical decreased the least (0.22 mm). The lingual bone thickness increased in the RME and SME groups (0.56 mm and 0.42 mm, respectively). The mesial root significantly increased in the RME group (0.52 mm) and in the SME group (0.40 mm), possibly due to incomplete root apex formation at T_1_ (prior to installation of expanders).

**Conclusions:**

Maxillary expansion (RME and SME) does not interrupt root formation neither shows first molar apical root resorption in juvenile patients. Although slightly larger in the RME group than SME group, both activation protocols showed similar buccal bone thickness and lingual bone thickness changes, without significant difference; and RME presented similar buccal cortical bone changes to SME.

## RESUMO

**Objetivo:**

o objetivo do presente trabalho foi realizar um estudo clínico randomizado comparando os efeitos da expansão rápida da maxila (ERM) e da expansão lenta da maxila (ELM). O comprimento radicular do primeiro molar superior permanente e o deslocamento dentro do alvéolo foram estudados por meio de imagens de tomografia computadorizada de feixe cônico (TCFC).

**Métodos:**

pacientes com deficiência maxilar transversal e faixa etária entre 7 e 10 anos foram incluídos no estudo. As crianças foram distribuídas em dois grupos: ERM (19 indivíduos com idade média de 8,60 anos) e ELM (13 indivíduos com idade média de 8,70 anos). Em ambos os grupos foi utilizado o aparelho expansor tipo Haas.

**Resultados:**

a cortical vestibular, a espessura do osso vestibular e a largura dentoalveolar sofreram redução nos dois grupos. No grupo ERM, a maior redução foi relacionada com a espessura do osso distal (1,26 mm), seguida pela espessura do osso mesial (1,09 mm), largura alveolar (0,57 mm) e, finalmente, pela cortical vestibular (0,19 mm). Já no grupo ELM, a espessura do osso mesial apresentou maiores valores de redução (0,87 mm), enquanto a cortical vestibular teve a menor redução (0,22 mm). A espessura do osso lingual aumentou nos dois grupos, sendo 0,56 mm no grupo ERM e 0,42 mm no grupo ELM. A raiz mesial aumentou significativamente nos grupos ERM (0,52 mm) e ELM (0,40 mm) - possivelmente, em função da rizogênese incompleta ainda em T_1_ (antes da instalação do aparelho).

**Conclusões:**

a expansão maxilar (ERM e ELM) não interrompe a formação ou leva à reabsorção radicular nos primeiros molares permanentes de pacientes jovens. Embora discretamente maiores no grupo ERM, as modificações na espessura do osso vestibular e lingual demonstradas pelos protocolos de ativação não apresentam diferenças significativas. O grupo ERM apresentou alterações semelhantes ao grupo ELM, em relação à cortical óssea vestibular.

## INTRODUCTION

Maxillary expansion has been used for more than 150 years^1^ and is a widely accepted procedure performed by orthodontists to correct posterior crossbite and transverse maxillary deficiency. To promote maxillary expansion, three treatment modalities are used today: rapid maxillary expansion (RME), slow maxillary expansion (SME) and surgically-assisted rapid palatal expansion (SARPE). Also, tooth-borne, bone-borne, tooth-tissue-borne, and hybrid (combination of two types) expanders are used to provide the maxillary expansion. The tooth-tissue-borne expander, which is recommended by Haas,^2^ is the most commonly used type.

Different rates of the screw activation can result in RME or SME.^3^ ^,^ ^4^ Using the jackscrew expander, RME is usually defined as two turns per day, while SME is defined as one turn every other day or at a greater interval.^5^ RME has been extensively used, and the greatest changes have been reported on the transverse plane (skeletally and dentally).^4^ But some limitations also have been reported, such as excessive tipping of anchorage teeth.^6^ Also, SME produces less tissue resistance around circummaxillary structures, improving bone formation, which theoretically should eliminate or reduce some limitations of the RME.^7^ ^-^ ^9^

It has been established that excessive tooth movements in the facial or buccal directions can lead to reductions in alveolar bone crest levels, bone dehiscence, and gingival recession.^10^ ^,^ ^11^ Histologic studies have shown that RME promotes root resorption on anchor teeth in patients who received RME treatment.^12^ ^-^ ^16^ Recent studies using cone-beam computed tomography (CBCT) demonstrated dehiscences and significant decreases in buccal bone thickness in patients treated with rapid palatal expanders.^17^ ^,^ ^18^

To provide comparisons between RME and SME using the Haas-type expander, analyzed by CBCT, this study was designed to evaluate maxillary first molar root length and tooth movement through the alveolus. It is important for the orthodontist to recognize if there is root resorption and a substantial decrease/increase in dentoalveolar bone thickness due to the two modalities of expansion. 

## MATERIAL AND METHODS

### Trial design and changes after trial commencement

This study was a randomized clinical trial conducted at the Federal University of Santa Catarina (UFSC, Brazil), approved by the ethics committee of this university (IRB# 1834, dated 04/25/2011). The sample included individuals from public schools in Florianópolis/SC, as well as patients seeking orthodontic treatment at the UFSC. The sample was divided into two groups: RME and SME. Informed consent was obtained from the parents of all patients who agreed to participate in this study.

### Participants and eligibility criteria

The inclusion criteria were as follows: transverse maxillary deficiency (posterior crossbite and dark buccal corridor), inter-transitory period of mixed dentition, ages between 7 and 10 years, and absence of metallic restorations on upper first molars. Patients were excluded if their CBCT images were not sufficiently clear to identify the landmarks, lack of proper activation of the appliance (patient did not follow the activation protocol), or exfoliation of their deciduous first molars during the expansion phase.

### Randomization and interventions

Fifty-nine patients agreed to participate in the study and were randomly divided into RME and SME groups. Microsoft Excel (version 2010, Microsoft, Seattle) was used to generate the randomization numbers. Thirty-two subjects remained in the study after the excluding criteria.

A Haas-type expander was cemented in all patients as recommended by Haas.^2^ Each appliance included a screw-type expander with maximum aperture of 11.0 mm (Dentaurum, Inspringen, Germany). The tooth-tissue-borne expanders were activated exactly 8 mm,^19^ according to the protocols of activations in both groups, for a total of 40 activations. At the end of activation, the appliances were stabilized with 0.12-mm ligature wires (Morelli, Sorocaba, Brazil).

The RME group consisted of 19 patients (13 girls and 6 boys, mean age of 8.60 years) who were treated by RME, with ½ turn (0.4 mm) per day and activated with a full turn on the first day. Total treatment time was three weeks. The SME group initially consisted of 13 patients (6 girls and 7 boys, mean age of 8.70 years) who were treated by SME, with a ½ turn (0.4 mm) per week (¼ turn on Tuesdays and ¼ on Fridays) and who were activated with a ½ turn on the first day, achieving total treatment time of 20 weeks. Patients were followed weekly to control the activation protocol. After expansion, the devices were stabilized with 0.12-mm wire and the Haas-type expander was used as a retainer for an additional six months after the initial activation in both groups.

### Records

**Figure 1:**
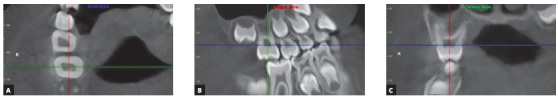
CBCT image orientation using the axial (A), sagittal (B), and coronal (C) views at first maxillary molar pulp chamber level.

All patients were subjected to CBCT between 1 and 7 days prior to installation of expanders (T_1_) and after six months of the initial activation (T_2_). An i-CAT machine (Imaging Sciences International, Hatfield, PA) was used to obtain CBCT images. The CBCT scans were performed at 120 Kv, 20 mA, and scan time of 14.7 seconds, with 0.25-mm isotropic voxels. The data for each patient were reconstructed with 0.5-mm slice thickness, and the DICOM files and the images were assessed by using Dolphin 3D Imaging v. 11.7 (Dolphin Imaging Systems, Chatsworth, CA). In this software, the orientation involved the following process (Fig 1):

1. The first step was to adjust the coronal and sagittal planes to intersect in the middle of first molar pulp chamber, as viewed on the axial plane.
2. After the coronal and sagittal planes were adjusted to intersect in the center of the tooth chamber on axial view, the axial plane was rotated so that the sagittal plane passed through the most mesial and distal aspects of the tooth.
3. In the final step of the orientation, sagittal and axial planes were adjusted to intersect at the lingual and buccal cemento-enamel junctions (CEJs).

**Figure 2:**
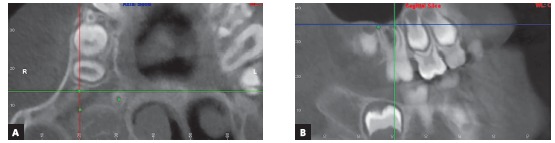
Identification of the root apex on the axial view (A) and double check position on the sagittal view (B).

After the orientation, six landmarks were digitized to measure the apical root length. Mesial, distal, and mesiopalatal cusps were digitized on the coronal view. To digitize the cusps, the coronal plane was moved forward and backward on the sagittal view until the most occlusal point of each cusp could be found in the coronal view. Each root apex was identified by moving the axial plane apically; it was digitized on the slice just before the root disappeared, and it was checked on the sagittal view (Fig 2).

**Figure 3:**
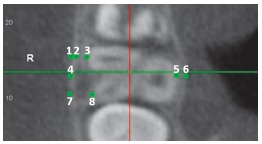
Axial view at buccal furcation of the first maxillary molar, to measure the linear distances Bcort (1-2), MBthick (1-3), Awidth (4-6), LBthick (5-6), DBthick (7-8).

Eight landmarks were made at the level of the buccal furcation of the first maxillary molar (Fig 3). Adjustments were made rotating the axial view to make the cortical buccal bone parallel to the sagittal line prior to digitizing the landmarks.

For each of the 14 landmarks, X, Y, and Z coordinates were produced. These coordinates were used to calculate the 8 distances of interest (Table 1). All landmarks were digitized twice, each one with an independent orientation in different days. Right and left sides of the same patient, with individual orientation, were performed at the same session. The average of the multiple digitalization was then used as the true value.

**Table 1:** Description of the eight variables.

Variable	Description
Bcort	Buccal cortical plate thickness: defined as the distance between the outer and inner borders of the buccal alveolar cortical plate in the area of the mesial root of the maxillary first molar
MBthick	Mesial bone thickness: shortest distance between the outer buccal alveolar cortical plate and the mesiobuccal root
Awidth	Alveolar width: measured from the outer limits of the buccal and lingual cortical plates, passing through the center of the maxillary first molar furcation
LBthick	Lingual bone thickness: shortest distance between the outer lingual alveolar cortical plate and the palatal root
DBthick	Distal bone thickness: shortest distance between the outer buccal alveolar cortical plate and the distobuccal root
M_Root	Mesial root length: distance from the mesiobuccal cusp to mesiobuccal root apex
D_Root	Distal root length: distance from the distobuccal cusp to distobuccal root apex
P_Root	Palatal root length: distance from the mesiopalatal cusp to palatal root apex

### Blinding

The same operator, who was unaware of the group to which each patient was assigned, performed all the measurements.

### Statistical analysis

Although the data presented normal distribution, non-parametric procedures were used to compare changes over time within groups (Wilcoxon signed rank) and to evaluate group differences (Mann-Whitney U) due to sample size. Percentiles were calculated for each measurement. Pearson correlations were used to relate the measures. Intraexaminer reliability was evaluated by using paired *t* tests for systematic and random errors between replicates. Systematic differences between the replicates were described with mean absolute differences ± standard errors (SE); differences were assessed using a paired Student’s *t* test. Random errors between replicates were quantified using the method error statistic (√ (Ʃ differences^2^/2n)).^20^ The data obtained from all measurements were processed with SPSS software (SPSS v. 22.0, IBM Corporation, USA).

## RESULTS

### Participant flow

A total of 59 patients (mean age = 8.18 years) were initially allocated into the groups. Twenty-seven patients were removed from the sample for several reasons. Two subjects from the RME group dropped out of the study during the expansion phase because they did not want to continue treatment. Four of the patients in the RME group and two in the SME group were removed because their CBCT images had not been taken within seven days of appliance removal. An additional three patients in the SME group were removed because their CBCT images were not clear. Two patients in the RME group and six in the SME group were removed due to exfoliation of the first deciduous molars during treatment. Three patients in the RME group and five in the SME group were removed because their appliances had not been properly activated (patients did not exactly follow the activation protocol). Patient recruitment initiated in July 2007 and finished in October 2011.

### Baseline data

Baseline information including sex, age and presence of clinical maxillary deficiency was gathered at the initial records. Both groups demonstrated similar baseline characteristics.

### Numbers analyzed for each outcome, estimation and precision, subgroup analyzes

Systematic intraexaminer reliability ranged between 0.058 mm and -0.667 mm. Three differences were statistically significant before treatment: distal root length and palatal root length on the right side, and alveolar width on the left side (Table 2). Method error ranged between 0.021 and 0.609 mm (Table 3).

**Table 2:** Intraexaminer reliability of M_Root, D_Root, P_Root, Bcort, MBthick, Awidth, LBthick, and DBthick.

	Systematic error		Random error
	Mean (mm)	SEM	Mean (mm)
M_Root			
Right	0.043	0.071	0.179
Left	-0.018	0.075	0.110
D_Root			
Right	-0.159	0.063*	0.515
Left	0.049	0.067	0.392
P_Root			
Right	-0.154	0.074*	0.208
Left	-0.047	0.066	0.609
Bcort			
Right	0.058	0.035	0.168
Left	0.017	0.033	0.028
MBthick			
Right	-0.041	0.038	0.212
Left	-0.012	0.038	0.117
Awidth			
Right	0.020	0.035	0.041
Left	-0.667	0.275*	0.149
LBthick			
Right	-0.006	0.031	0.121
Left	0.023	0.032	0.022
DBthick			
Right	0.035	0.035	0.021
Left	-0.044	0.031	0.195

**Table 3:** Pretreatment comparison of the dentoalveolar thickness between right and left sides.

	Measurements	Right side			Left side			P value
		Percentiles			Percentiles			
		25	50	75	25	50	75	
RME	Bcort	0.85	1.03	1.34	0.84	0.98	1.14	0.295
	MBthick	2.14	2.51	3.59	1.88	2.34	2.80	0.023*
	Awidth	14.94	15.35	16.13	14.59	15.49	16.07	0.276
	LBthick	1.44	1.74	1.90	1.39	1.74	1.93	0.695
	DBthick	2.29	2.89	3.90	2.33	3.15	3.57	0.968
SME	Bcort	0.70	0.83	0.88	0.63	0.68	0.94	0.363
	MBthick	1.86	2.59	2.88	1.92	2.36	2.64	0.402
	Awidth	14.30	14.90	15.43	14.25	15.14	15.94	0.363
	LBthick	1.11	1.25	1.61	1.06	1.28	1.54	0.625
	DBthick	2.14	2.53	3.08	2.53	2.83	3.19	0.196

*Statistically significant (p < 0.05).

There was only one statistically significant difference between the right and left sides (Tables 3 and 4): bone thickness at mesiobuccal root was different (*p*= 0.023) between sides at pretreatment in the RME group. Due to only this one difference and the similar pattern trends, the right side was selected to make the comparison between RME and SME groups.

**Table 4:** Pretreatment comparison of the dental root length between right and left sides.

	Measurements	Right side			Left side			P value
		Percentiles			Percentiles			
		25	50	75	25	50	75	
RME	M_Root	16.08	17.07	18.08	16.24	17.33	18.23	0.825
	D_Root	16.02	16.95	17.69	15.99	16.70	17.85	0.952
	P_Root	18.26	19.33	20.31	17.63	19.09	20.27	0.546
SME	M_Root	17.15	17.70	18.46	17.48	18.14	18.35	0.196
	D_Root	16.82	17.63	18.40	17.22	17.92	18.71	0.116
	P_Root	19.37	20.08	20.54	19.55	20.30	20.82	0.507

Transverse dentoalveolar pretreatment showed differences between the two groups in the right side (Table 5). Buccal cortical and lingual alveolar thicknesses were thicker in the RME group than in the SME group. Bcort was 0.2 mm thicker and LBthick 0.36 mm thicker in the RME group, compared to SME group. No differences were significant in root length between groups before treatment (Table 6).

**Table 5:** Pretreatment comparison of dentoalveolar thickness between RME and SME groups.

Measurements	RME			SME			P value
	Percentiles			Percentiles			
	25	50	75	25	50	75	
Bcort	0.85	1.03	1.34	0.70	0.83	0.88	0.017*
MBthick	2.14	2.51	3.59	1.86	2.59	2.88	0.347
Awidth	14.94	15.35	16.13	14.30	14.90	15.43	0.058
LBthick	1.44	1.74	1.90	1.11	1.25	1.61	0.025*
DBthick	2.29	2.89	3.90	2.14	2.53	3.08	0.167

*Statistically significant (p < 0.05).

**Table 6:** Pretreatment comparison of dental root length between RME and SME groups.

Measurements	RME			SME			P value
	Percentiles			Percentiles			
	25	50	75	25	50	75	
M_Root	16.08	17.07	18.08	17.15	17.70	18.46	0.205
D_Root	16.02	16.95	17.69	16.82	17.63	18.40	0.084
P_Root	18.26	19.33	20.31	19.37	20.08	20.54	0.150

Related to treatment, all five dentoalveolar measurements showed significant differences within groups (Table 7). Once the first maxillary molars moved buccally due to maxillary expansion, the buccal cortical, the buccal bone thicknesses, and the dentoalveolar width decreased in both groups. In the RME group the greatest decrease was related to distal bone thickness (1.26 mm) followed by mesial bone thickness (1.09 mm), alveolar width (0.57 mm), and the buccal cortical (0.19 mm). In the SME group the mesial bone thickness decreased the most (0.87 mm) and the buccal cortical bone decreased the least (0.22 mm). The lingual bone thickness increased in the RME and SME groups (0.56 mm and 0.42 mm, respectively).

**Table 7:** Comparison of dentoalveolar thickness changes (T2-T1) within and between RME and SME groups.

	RME			Within RME	SME			Within SME	RME vs. SME
Measurements	Percentiles			P value	Percentiles			P value	P value
	25	50	75		25	50	75		
Bcort	-0.42	-0.19	0.10	0.042*	-0.36	-0.22	-0.08	0.008*	0.788
MBthick	-1.34	-1.09	-0.89	< 0.001*	-1.71	-0.87	-0.72	0.001*	0.502
Awidth	-1.52	-0.57	-0.33	0.001*	-0.93	-0.38	-0.05	0.033*	0.266
LBthick	0.13	0.56	1.08	0.002*	0.26	0.42	1.07	0.004*	0.863
DBthick	-1.66	-1.26	-0.78	< 0.001*	-1.54	-0.70	-0.51	0.001*	0.192

*Statistically significant (p < 0.05).

Although no significant differences were found between groups, the mesial root length was the only measurement that showed a difference due to maxillary expansion within groups in RME and SME groups (Table 8). The mesial root increased significantly 0.52 mm (*p*= 0.003) in the RME group and 0.40 mm (*p*= 0.013) in the SME group.

**Table 8:** Comparison of dental root length changes (T_2_-T_1_) within and between RME and SME groups.

Measurements	RME			Within RME	SME			Within SME	RME vs. SME
	Percentiles			P value	Percentiles			P value	P value
	25	50	75		25	50	75		
M_Root	0.12	0.52	1.15	0.003*	0.11	0.40	0.94	0.013*	0.604
D_Root	-0.41	0.19	0.99	0.159	-0.22	0.28	0.59	0.133	0.878
P_Root	-0.33	0.32	0.94	0.107	-0.21	0.16	0.45	0.196	0.388

*Statistically significant (p < 0.05).

Pearson correlations showed that changes in buccal movement of the molars are positively correlated to buccal bone thickness and negatively correlated to lingual bone thickness (Table 9). Bone thickness at mesial root is moderate high positively correlated to bone thickness at distal root (r = 0.697) and alveolar width (r = 0.566). Also, mesial bone thickness is moderate positively correlated to buccal cortical thickness (r = 0.502). Decreasing the bone thickness at distal root increases the lingual bone thickness (r = -0.361). Changes in the distal root have more positively correlation with palatal root changes (r = 0.591) than changes in the mesial root (r = 0.389). Root changes were not correlated to changes in buccal bone thickness, lingual bone thickness, or alveolar width.

**Table 9:** Pearson correlations of the changes in the dentoalveolar thickness and dental root length (T_2_-T_1_).

Measurements	Bcort	MBthick	Awidth	LBthick	DBthick	M_Root	D_Root	P_Root
Bcort	1	0.502*	0.012	- 0.179	0.172	0.113	- 0.098	- 0.1
MBthick		1	0.566*	- 0.332	0.697*	- 0.118	- 0.054	- 0.088
Awidth			1	0.294	0.454*	- 0.349	- 0.084	- 0.198
LBthick				1	- 0.361*	- 0.329	- 0.278	- 0.454*
DBthick					1	- 0.111	0.112	< 0.001
M_Root						1	0.389*	0.17
D_Root							1	0.591*
P_Root								1

*Statistically significant (p < 0.05).

## DISCUSSION

Buccal bone thickness decreased substantially during treatment. The RME group showed slightly but not significant greater decreases in the buccal bone thickness than the SME group (0.6 mm and 0.2 mm at distal root level and mesial root levels, respectively). The decreases were smaller than previously reported.^18^ ^,^ ^21^ The smaller losses in buccal bone thickness in the present study could also be attributed to increased anchorage due to appliance design of the Haas-type expander. According to Haas, the acrylic pad helps to reinforce the anchorage for greater orthopedic and smaller dental responses during maxillary expansion.^2^ Using a small sample size, Oliveira et al^22^ showed greater orthopedic movement in Haas-type than Hyrax-type expander, but the same amount of molar tipping. Additionally, Weissheimer et al^19^ did not support the theory that tooth-tissue-borne and tooth-borne expanders have differences in dentoalveolar and molar angulation changes, at least regarding the immediate results of the expansion. Differences between studies might be due to different appliances design, amount of activation, and time frame.

The decrease in the buccal bone thickness was approximately twice as great as the increase in lingual bone thickness, due to treatment causing thinner dentoalveolar width at molar level. Longitudinal arch width measurements have shown greater increases in palatal alveolar widths than in buccal alveolar widths, decreasing approximately 0.25 mm per side between 7.6 and 10.3 years of age in untreated subjects.^23^ Previous studies showed similar decreases in buccal bone thickness and increases in lingual bone thickness due to maxillary expansion.^17^ ^,^ ^21^ Also, Corbridge et al^21^ showed that alveolar width increased slightly. The authors suggested that the alveolar bone was partially adapting to the expansion and maybe the present study could not show the same effect due to short period of time between evaluations. It is suggested that there is less molar buccal tipping when maxillary expansion is performed with Haas-type expander, due to acrylic pads resting on the palatal shelves, but literature is not consistent about it.^17^ ^,^ ^19^ ^,^ ^22^ Although the present study did not evaluate the inclination, it seems reasonable that tipping can influence the amount of buccal and lingual bone thickness.

Independently of the type of maxillary expansion (RME or SME), the treatments were able to move teeth through the cortical plate. Buccal bone cortical plate decreased similarly in both groups (approximately 0.2 mm), but rapid and slow maxillary expansions showed different tooth movement within bone. RME showed greater buccal movement of the distal buccal root than mesial buccal root, while SME showed the opposite pattern (Fig 4). Corbridge et al^21^ evaluated slow maxillary expansion using quad-helix appliance and found that the mesial buccal root moved more toward to the buccal cortical alveolar bone than the distal root. Also, RME showed greater tooth movement within alveolar bone. It is probable that in the RME group, the greater amount of tooth movement could be generated by larger molar inclination, as reported in a previous study.^24^ This suggests that the alveolar bone partially adapts to the treatment.

Maxillary expansion with Haas-type expander did not show first molar apical root resorption in juvenile patients. Mesial roots showed an increase in length after expansion in both RME and SME groups. Due to the patients’ ages, the molar root apices were not closed, allowing increases in the root length. It is known that the maxillary first molar has the root completed at approximately 10 years of age,^25^ and this could explain why the root length showed increases. Evaluating cleft lip and palate patients between 8 and 15 years of age, Cardinal et al^26^ showed that the rapid palatal expansion did not interrupt maxillary first molar roots formation. The authors showed that the palatal roots increased almost 0.5 mm in subjects presenting open apex.^26^ Reporting five adult cases treated with maxillary expansion, Handelman^27^ showed minimal root resorption in 2-D radiographs. Using CBCT, Baysal et al^28^ found a decrease in molar root volumes after RME. The problem involving volume analysis is that it is not possible to precisely evaluate where the root resorption occurred. Most of the previous studies that evaluated root resorption due to maxillary expansion have related resorption to the buccal surface of premolar roots that were used as anchorage to the maxillary expansion.^13^ ^,^ ^29^ ^,^ ^30^ Unfortunately, the present study did not have an untreated control group, due to ethical concerns. The observation of untreated patients would be important to differentiate natural root lengthening from the changes derived from treatment, especially in the SME group, where the opening of the screw extended for five months.

RME group showed the shortest root (distal root on the left side, with median of 16.70 mm), and SME group showed the longest root (right palatal root on the right side, with median of 20.30 mm) at pretreatment, and root lengths were longer after treatment. On average, maxillary first molar roots varied from 16 mm to 24 mm in length.^31^ Normally, maxillary first molar mesial root length is approximately 19.5 mm, distal root length is approximately 19.2 mm, and palatal root is 20.5 mm.^32^ In subjects presenting open apexes before rapid palatal expansion, Cardinal et al^26^ showed that the palatal root length increased after the treatment, presenting 19.3 mm. The lack of untreated subjects as a control group makes it difficult to state that roots became shorter than expected, because the length is similar to the literature mean.

The buccal displacement of the maxillary first permanent molars in both protocols, decreasing the buccal thickness, should be regarded as a consequence of the palatal expansion procedure. To some extent, subjects probably will present periodontal sequelae to the anchorage teeth of the palatal expander, making them more susceptible to periodontal problems in the long term.^33^ From a periodontal point of view, maxillary expansion perhaps could be performed in the deciduous or early mixed dentition, because the eruption of permanent teeth can minimize the periodontal effects produced by rapid or slow maxillary expansion.

## CONCLUSION

1. Maxillary expansion (RME and SME) does not interrupt root formation neither show first molar apical root resorption in juvenile patients (7-10 years of age).
2. Although slightly larger in the RME group than SME group, both activation protocols showed similar buccal bone thickness and lingual bone thickness changes, without significant difference.
3. RME presents similar buccal cortical bone changes than SME.

## References

[1] Angell EH (1860). Treatment of irregularity of the permanent or adult teeth. Dent Cosmos.

[2] Haas AJ (1970). Palatal expansion just the beginning of dentofacial orthopedics. Am J Orthod.

[3] Lagravere MO, Major PW, Flores-Mir C (2005). Skeletal and dental changes with fixed slow maxillary expansion treatment a systematic review. J Am Dent Assoc.

[4] Lagravere MO, Heo G, Major PW, Flores-Mir C (2006). Meta-analysis of immediate changes with rapid maxillary expansion treatment. J Am Dent Assoc.

[5] Huynh T, Kennedy D, Joondeph D, Bollen AM (2009). Treatment response and stability of slow maxillary expansion using Haas, hyrax, and quad-helix appliances: a retrospective study. Am J Orthod Dentofacial Orthop.

[6] Capelozza L, Cardoso J, da Silva OG, Ursi WJ (1996). Non-surgically assisted rapid maxillary expansion in adults. Int J Adult Orthodon Orthognath Surg.

[7] Hicks EP (1978). Slow maxillary expansion a clinical study of the skeletal versus dental response to low-magnitude force. Am J Orthod.

[8] Bell RA (1982). A review of maxillary expansion in relation to rate of expansion and patient's age. Am J Orthod.

[9] Mew J (1983). Relapse following maxillary expansion a study of twenty-five consecutive cases. Am J Orthod.

[10] Steiner GG, Pearson JK, Ainamo J (1981). Changes of the marginal periodontium as a result of labial tooth movement in monkeys. J Periodontol.

[11] Engelking G, Zachrisson BU (1982). Effects of incisor repositioning on monkey periodontium after expansion through the cortical plate. Am J Orthod.

[12] Isaacson RJ, Ingram AH (1964). Forces produced by rapid maxillary expansion. Angle Orthod.

[13] Langford SR (1982). Root resorption extremes resulting from clinical RME. Am J Orthod.

[14] Moss JP (1968). Rapid expansion of the maxillary arch Part I. J Clin Orthod.

[15] Moss JP (1968). Rapid expansion of the maxillary arch Part II. Indications for rapid expansion. JPO J Pract Orthod.

[16] Timms DJ (1968). An occlusal analysis of lateral maxillary expansion with midpalatal suture opening. Dent Pract Dent Rec.

[17] Garib DG, Henriques JF, Janson G, Freitas MR, Fernandes AY (2006). Periodontal effects of rapid maxillary expansion with tooth-tissue-borne and tooth-borne expanders: a computed tomography evaluation. Am J Orthod Dentofacial Orthop.

[18] Rungcharassaeng K, Caruso JM, Kan JYK, Kim J, Taylor G (2007). Factors affecting buccal bone changes of maxillary posterior teeth after rapid maxillary expansion. Am J Orthod Dentofacial Orthop.

[19] Weissheimer A, Menezes LM, Mezomo M, Dias DM, Lima EMS, Rizzatto SMD (2011). Immediate effects of rapid maxillary expansion with Haas-type and hyrax-type expanders: A randomized clinical trial. Am J Orthod Dentofacial Orthop.

[20] Dalhberg G (1940). Statistical methods for medical and biological students.

[21] Corbridge JK, Campbell PM, Taylor R, Ceen RF, Buschang PH (2011). Transverse dentoalveolar changes after slow maxillary expansion. Am J Orthod Dentofacial Orthop.

[22] Oliveira NL, Silveira AC, Kusnoto B, Viana G (2004). Three-dimensional assessment of morphologic changes of the maxilla: a comparison of 2 kinds of palatal expanders. Am J Orthod Dentofacial Orthop.

[23] Hesby RM, Marshall SD, Dawson DV, Southard KA, Casko JS, Franciscus RG, Southard TE (2006). Transverse skeletal and dentoalveolar changes during growth. Am J Orthod Dentofacial Orthop.

[24] Brunetto M, Andriani JSP, Ribeiro GLU, Locks A, Correa M, Correa LR (2013). Three-dimensional assessment of buccal alveolar bone after rapid and slow maxillary expansion a clinical trial study. Am J Orthod Dentofacial Orthop.

[25] Proffit WR, Fields HW, Sarver DM (2007). Contemporary Orthodontics.

[26] Cardinal L, Zimermann GR, Mendes FM, Andrade I, Oliveira DD, Dominguez GC (2018). The impact of rapid maxillary expansion on maxillary first molar root morphology of cleft subjects. Clin Oral Investig.

[27] Handelman CS (1997). Nonsurgical rapid maxillary alveolar expansion in adults a clinical evaluation. Angle Orthod.

[28] Baysal A, Karadede I, Hekimoglu S, Ucar F, Ozer T, Veli I (2012). Evaluation of root resorption following rapid maxillary expansion using cone-beam computed tomography. Angle Orthod.

[29] Barber AF, Sims MR (1981). Rapid maxillary expansion and external root resorption in man: a scanning electron microscope study. Am J Orthod.

[30] Erverdi N, Okar I, Kücükkeles N, Arbak S (1994). A comparison of two different rapid palatal expansion techniques from the point of root resorption. Am J Orthod Dentofacial Orthop.

[31] Ford TRP, Rhodes JS, Ford HEP (2002). Endodontics: problem-solving in clinical practice.

[32] Caliskan MK, Pehlivan Y, Sepetçioglu F, Türkün M, Tuncer SS (1995). Root canal morphology of human permanent teeth in a Turkish population. J Endod.

[33] Greenbaum K, Zachrisson B (1982). The effect of palatal expansion therapy on the periodontal supporting tissues. Am J Orthod.

